# Role of endosomal toll-like receptors in immune sensing of *Klebsiella pneumoniae*

**DOI:** 10.3389/fimmu.2025.1538425

**Published:** 2025-04-03

**Authors:** Giuseppe Valerio De Gaetano, Agata Famà, Germana Lentini, Francesco Coppolino, Mario Venza, Isabella Venza, Pasqualina Laganà, Alessia Berbiglia, Federica Grasso, Luigi Fiore, Giuseppe Teti, Concetta Beninati

**Affiliations:** ^1^ Department of Human Pathology “Gaetano Barresi”, University of Messina, Messina, Italy; ^2^ Department of Biomedical and Dental Sciences and Morphological and Functional Images, University of Messina, Messina, Italy; ^3^ Department of Clinical and Experimental Medicine, University of Messina, Messina, Italy; ^4^ Scylla Biotech Srl, Messina, Italy

**Keywords:** gram negative bacteria, pneumonitis, pattern recognition receptors, neutrophils, cytokines, bacterial RNA

## Abstract

*Klebsiella pneumoniae* is the causative agent of a wide range of antibiotic-resistant infections, including nosocomial pneumonia and neonatal sepsis. We investigate here the mechanisms underlying innate immune recognition of this pathogen by focusing on the role of endosomal Toll-like receptors (TLRs), which sense prokaryotic nucleic acids, in comparison with TLR4, which recognizes the cell-wall lipopolysaccharide component. Lack of functional endosomal TLRs made mice more susceptible to pulmonary infection by *K. pneumoniae*, in association with reduced production of proinflammatory and chemotactic cytokines and reduced neutrophil recruitment to the lung. This phenotype was as severe as that of TLR4-deficient mice and only moderately milder than that of mice lacking the TLR adaptor MyD88. Notably, macrophages lacking at the same time TLR7, 9 and 13 were more defective than those lacking only TLR9 in their ability to produce proinflammatory cytokines, suggesting a role for the RNA sensing TLR7 and 13 receptors in *K. pneumoniae* recognition. Collectively, our results unveil the presence of an integrated system of DNA and RNA sensing TLRs that cooperates with TLR4 in immune detection and clearance of *K. pneumoniae*. These data may be useful to devise alternative therapeutic approaches aimed at stimulating responses against antibiotic-resistant *K. pneumoniae* strains.

## Introduction

*Klebsiella* species are ubiquitous Gram-negative microorganisms found in the environment, including water and soil, as well as in association with animals (1, 2). *Klebsiella pneumoniae*, an opportunistic encapsulated human pathogen, frequently colonizes the gastrointestinal mucosa and the oropharynx without causing disease. However, from these sites, *K. pneumoniae* can spread to other tissues, leading to severe conditions including urinary tract infections (UTIs), pneumonia, meningitis and septicemia (3–5). *K. pneumoniae* also frequently colonizes medical devices, being one of the most commonly isolated nosocomial pathogens in intensive care units (4, 5). An alarming proportion of *K. pneumoniae* clinical isolates is highly resistant to a wide variety of antibiotics, such as fluoroquinolones, third-generation cephalosporins, aminoglycosides and carbapenems, thus severely limiting available therapeutic options (4). Furthermore, recent studies suggest that emerging hypervirulent *K. pneumoniae* clones have simultaneously acquired virulence and antimicrobial genes, raising the potential for untreatable infections even in healthy individuals (6–8). In addition, *K. pneumoniae* can finely regulate the expression of virulence factors and antibiotic resistance genes through Two-Component Systems (TCSs) capable of sensing and responding to environmental signals (9–12). Due to these factors, the World Health Organization (WHO) recently identified *K. pneumoniae* as a critical public health threat and a major antimicrobial resistant (AMR) pathogen (13, 14), highlighting an urgent need to develop alternative therapeutic strategies, such as those that enhance host defenses. Defenses against *K. pneumoniae* and other microbes rely on the host’s ability to recognize invading pathogens through pattern-recognition receptors (PRRs) that are primarily found in cells of the mononuclear phagocyte system (such as monocytes, macrophages, and dendritic cells) as well as in granulocytes, including neutrophils. PRRs are strategically located in subcellular compartments where they detect conserved microbial components, known as pathogen-associated molecular patterns (PAMPs), and initiate intracellular signaling pathways that activate or enhance the host’s antimicrobial defenses (15). Toll-like receptors (TLRs) are one of the best-characterized classes of PRRs and are highly conserved across a wide range of species, from invertebrates to vertebrates (16–18). Upon recognition of microbial PAMPs, TLRs trigger complex downstream signaling pathways leading to the production and release of cytokines and chemokines that orchestrate a variety of innate and adaptive responses (19). Signaling molecules that are sequentially engaged by TLRs include the adaptor MyD88 (myeloid differentiation primary response gene 88), which leads to transcription of anti-microbial genes through the activation of mitogen-activated protein kinases, nuclear factor-κB and interferon releasing factors (20). In addition, TLR3 and TLR4 can activate MyD88-independent pathways that require the adaptor molecule TRIF (TIR domain-containing adaptor inducing interferon-β) (21).

TLR4, a cell surface-exposed receptor, has been historically identified as the most important TLR for the recognition of *K. pneumoniae* (22–25), due to its capacity to sense lipopolysaccharide (LPS), the major component of the outer membrane of Gram-negative bacteria (26). TLR2, an additional surface-exposed receptor, has also been reported to play a role in anti-*K. pneumoniae* defenses (27, 28). Finally, the endosomal receptor TLR9, which can sense unmethylated CpG-rich DNA motifs that are abundant in prokaryotic genomes, also participates in anti-*K. pneumoniae* defenses (20). Interestingly, in dendritic cells, TLR9 can cooperate with TLR4 to maximally amplify the production of IL-23 and IL-17 (22). Other *in vivo* studies focused on the involvement of MyD88 (which mediates signaling by all TLRs except TLR3) or TIRAP (an essential adaptor for TLR1, TLR2, TLR4 and TLR6 signaling), confirming the important role of TLR-initiated pathways in *K. pneumoniae* recognition (21, 29).

Although recent studies highlighted a crucial role of RNA-sensing TLRs, such as TLR7 and 13, in the recognition of Gram-positive bacterial pathogens (30–33), little is known of the functions of these receptors in the context of infection by *K. pneumoniae* or other Gram-negative pathogens. In the present study, by using mouse strains with defective TLR signaling, we demonstrate that endosomal receptors play a major role in sensing the presence of *K. pneumoniae* and in sustaining the production of a distinctive set of proinflammatory and chemotactic cytokines, particularly IL-12p70. We find that absence of nucleic acid-sensing TLR function is associated with markedly enhanced susceptibility to lung infection due to defective neutrophil recruitment. Moreover, *K. pneumoniae* RNA was found to be more potent than DNA in inducing cytokine production in macrophages, primarily through its ability to engage TLR7 and 13. These findings may provide valuable insights for the development of alternative therapeutic strategies that target innate immune receptors, offering a potential approach for treating infections caused by antibiotic-resistant *K. pneumoniae*.

## Materials and methods

### Mouse and bacterial strains

Wild type (WT) C57BL/6 female mice were purchased from Charles River Laboratories (Charles River, Calco, Italy). Mice lacking MyD88, TLR2, TLR3, TLR4, TLR7 and TLR9 on a C57BL/6 background were a generous gift of Shizuo Akira (from Osaka University, Suita, Japan). TLR13^-/-^ mice were obtained by intercrossing TLR13^+/-^ animals generated at the University of California, Davis Mouse Biology Program (www.mousebiology.org) and provided by the Knockout (KO) Mouse Project Repository (www.komp.org), as previously described (32). Triple KO mice simultaneously lacking TLR7, 9 and 13 have also been previously described (32, 33). Mice bearing the H412R mutation of UNC93B1 (or “3d” mice) were donated by Bruce Beutler (University of Texas Southwestern Medical Center, Texas). Individually ventilated cages were used to house all mice strains under specific pathogen-free conditions. *K. pneumoniae* AC133 (34) was used throughout the present investigation. Bacteria were grown to the mid-log phase in Luria Bertani broth, washed three times in nonpyrogenic phosphate-buffered saline (PBS) (0.01 M phosphate, 0.15 M NaCl [pH 7.4]; Thermo Fisher Scientific), and resuspended to the appropriate concentration in Luria Bertani broth before intranasal challenge (or in PBS before addition to phagocyte cultures) at the indicated multiplicities of infection (MOI, see below).

### Mouse model of *K pneumoniae* infection

Six-week-old female mice were anesthetized by the intraperitoneal injection of tiletamine-zolazepam (0.1 mg/mouse) and xylazine (0.16 mg/mouse) and inoculated intranasally (i.n.) with 20 µl of the bacterial suspension (1x10^7^ CFU/mouse). Clinical conditions were evaluated every 12h for 10 days after inoculation. Animals with irreversible signs of disease, as assessed by a scoring system based on predefined clinical criteria (34), were humanely euthanized using cervical dislocation. To study the ability of bacteria to grow *in vivo*, the animals were euthanized at the indicated times after challenge and transcardially perfused with PBS (20 ml), prior to lung collection, as previously described (30). To obtain lung homogenates, the organs were minced and enzymatically digested with collagenase, as described previously (30). Bacterial CFU were measured in organ homogenates by plating serial dilutions on agar plates by standard techniques, while neutrophil numbers were determined by flow cytometry as described below.

### Isolation of macrophages and neutrophils

3d, MyD88^-/-^, TLR4^-/-^ and TLR2^-/-^ mice were used as source of bone marrow-derived and bronchial washing cells that were isolated as previously described (34). Briefly, bone marrow cells were obtained from the extremities of the long bones after cutting off epiphyses through centrifugation at 400xg for 15 min, washed and resuspended in PBS. To obtain neutrophils, cells were centrifuged on a density Percoll gradient, as described (33). To obtain macrophages, marrow cells were cultured for 6 to 7 days in RPMI 1640 supplemented with heat-inactivated 10% FCS, penicillin (50 IU/ml), streptomycin (50 g/ml) and macrophage colony-stimulating factor (M-CSF; 100 ng/ml) or granulocyte-macrophage colony-stimulating factor (GM-CSF; 20 ng/ml), both from PeproTech. Frozen bone marrow derived macrophages originally obtained from TLR3^-/-^, TLR7^-/-^, TLR9^-/-^, TLR13^-/-^ and TLR7/9/13^-/-^ mice (33) were thawed and cultured in the presence of GM-CSF to obtain GM-CSF-polarized macrophages. To obtain alveolar macrophages (AM), the lungs of WT, 3d, MyD88^-/-^, TLR4^-/-^ or TLR2^-/-^ mice were lavaged with an intratracheal catheter with PBS supplemented with 1mM EDTA. The bronchoalveolar lavage fluid was centrifuged at 300xg for 10 min and cells were resuspended to a cell concentration of 5x10^6^/ml in RPMI 1640 supplemented with heat-inactivated 10% FCS, penicillin (50 IU/ml), streptomycin (50 g/ml), and 100 ng/ml M-CSF. Isolated cells were incubated at 37°C with 5% CO_2_ for 24 h and three washes were done to gently remove nonadherent cells.

### Cell stimulation

Macrophages were infected with PBS suspensions of *K. pneumoniae* at the indicated MOIs. A low-speed centrifugation step (400xg for 10 min) was carried out to facilitate interactions between bacteria and macrophages. After a 1 h incubation at 37°C with 5% CO_2_, gentamycin (100 μg/ml) was subsequently added to infected cells for killing extracellular bacteria, as previously described (35–38). Control wells were stimulated with *Escherichia coli* K-12 ultrapure LPS (InvivoGen). Cell culture supernatants were then collected at various times after infection and stored at 80°C for cytokine measurements. In selected experiments, bone marrow-derived macrophages (BMDMs) were stimulated with DNA and RNA extracted from *K. pneumoniae*. Briefly, bacterial nucleic acids were extracted and purified as previously described (33) and complexed with N-[1-(2,3-dioleoyloxy) propyl]-N, N, N-trimethylammonium methyl-sulfate (DOTAP) before cell stimulation, as previously described (32, 33, 39).

### Phagocytic killing assay

For phagocytosis/killing assays, WT and 3d macrophages (5x10^5^ cells/well) were infected with *K. pneumoniae* (1x10^7^ CFU/well) and incubated for 1 h at 37°C with 5% CO_2_. After three washes with PBS and killing of extracellular bacteria through the addition of medium containing gentamicin (100 μg/ml), macrophages were washed, detached, and lysed with 0.025% Triton X-100 to release intracellular bacteria. Recovered bacteria were plated on agar plates for CFU counts.

### Cytokine measurements

Commercial enzyme-linked immunosorbent assay (ELISA) kits were used to measure cytokine and chemokines levels in lung homogenates and culture supernatants, as described (40). Keratinocyte-derived chemokine (KC; Cxcl1), macrophage inflammatory protein 2 (MIP-2; Cxcl2), IL-1β, TNF-α, IL-12 p70, IFN-β, and IFN-ℽ were determined in triplicate with the following ELISA kits (all from R&D Systems): CXCL1/KC Quantikine, DuoSet CXCL2/MIP-2, IL-1β/IL-1F2 Quantikine, DuoSet TNF-α, DuoSet IL-12 p70, IFN-β DuoSet and Duoset IFN-ℽ. The lowest detection limits of these assays were, respectively, 15.6, 15.6, 12.5, 16.0, 39.1, 12.5, and 31.3 pg/ml.

### Real-time PCR measurements

Total RNA was extracted from macrophages (1x10^7^) using the RNeasy mini kit (Qiagen) according to the manufacturer’s instructions as previously described (41, 42). After determining RNA quality and yield by agarose gel electrophoresis and spectrophotometry, cDNA was synthesized using the Moloney murine leukemia virus reverse transcriptase kit (M-MLV RT; Invitrogen), and expression of cytokine encoding genes was determined by qPCR by the delta-delta threshold cycle (ΔΔCT) method normalized against the β-actin housekeeping gene, as described (30).

### Flow cytometry

Neutrophils were enumerated in lung homogenates during *K. pneumoniae* infection by flow cytometry on a FACS Canto II flow cytometer (BD Biosciences) using BD Trucount beads (BD Biosciences), as previously described (33). Briefly, single cell suspensions obtained from lung homogenates were incubated for 20 min with mouse Fc block (rat anti-mouse CD16/CD32 clone 2.4G2, BD Pharmingen) and then stained for 20 min with antibodies directed against Ly6G (phycoerythrin [PE]–rat anti-mouse Ly6G clone 1A8; BD Pharmingen) or with an isotype control monoclonal antibody. Data analysis was performed using Flowing Software 2.5.1.

### Reactive oxygen species measurement

CellROX Deep Red Flow Cytometry Assay Kit (Thermo Fisher Scientific) was used to measure bacteria-induced reactive oxygen species (ROS) production, according to the manufacturer’s instructions. Briefly, bone marrow-derived neutrophils from WT or immune-deficient mice were seeded at a concentration of 5 x 10^5^ per well in RPMI 1640 supplemented with 10% FCS and stimulated for 30 min with the indicated doses of *K. pneumoniae*. Then, stimulated neutrophils were stained with the CellROX fluorescent reagent (5µM) for 30 min at 37°C. Cells were washed three times, fixed with 3.7% formaldehyde for 15 min and analyzed with a BD FACSCanto II instrument. Flowing Software 2.5.1 was used for data analysis.

### Statistical analysis

Differences in CFU counts and in cytokine or chemokine levels were assessed by Mann-Whitney test. Survival data obtained in *in vivo* assays were analyzed by the log-rank (Mantel-Cox) test. Differences were considered statistically significant when p values were less than 0.05. Statistical analyses were performed with GraphPad Prism 8.2.0 (GraphPad Software, Inc., San Diego, CA).

### Ethics statement

All animal studies were performed in strict accordance with the European Union guidelines for the use of laboratory animals. The procedures were approved by the Animal Welfare Committee of the University of Messina (OPBA) and by the Ministero della Salute of Italy (permit numbers 785/2018-PR and 1023/2024-PR).

## Results

### Mice with defective endosomal TLR function are impaired in anti-*K pneumoniae* defenses

**Figure 1:**
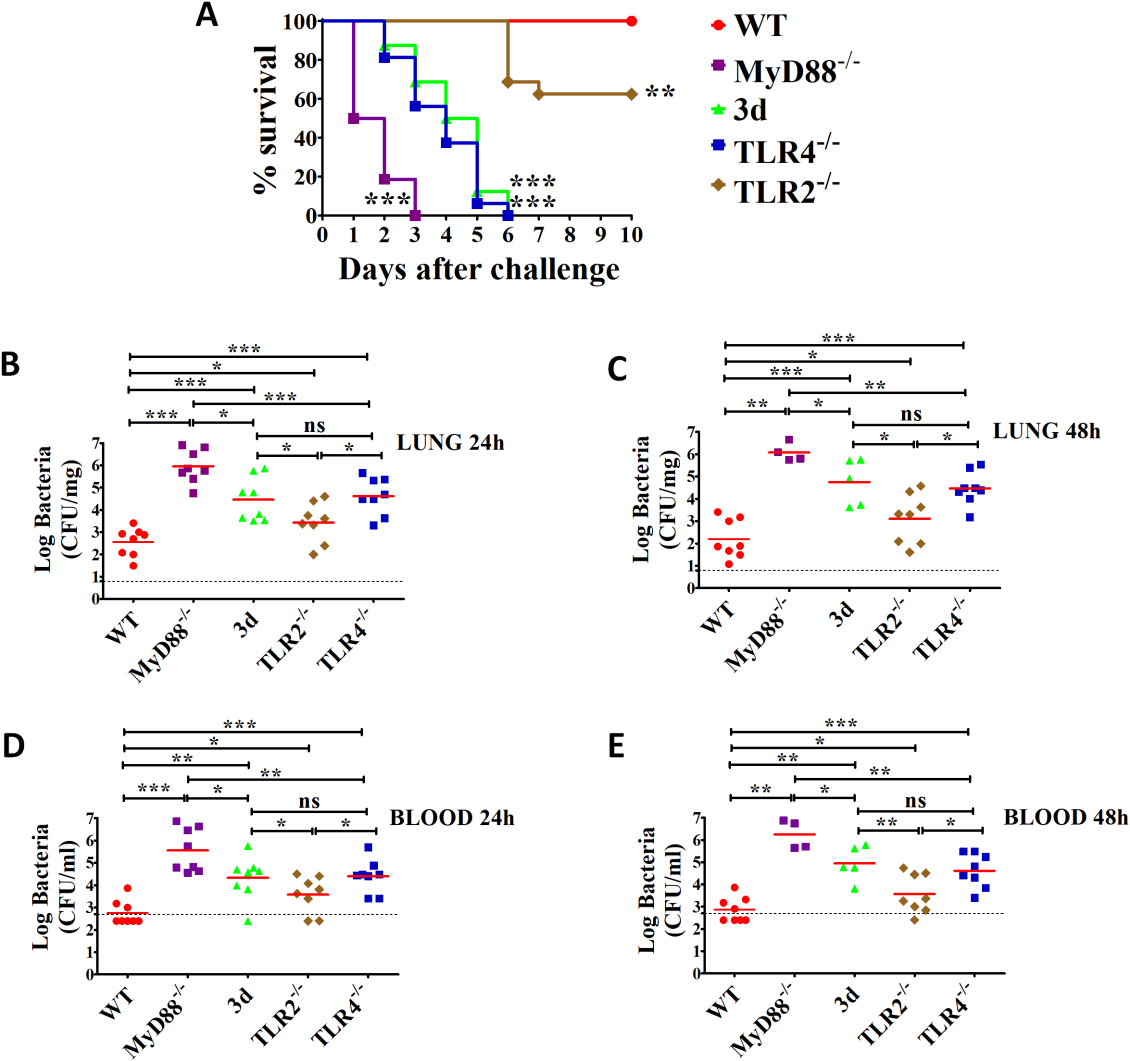
Mice with non-functional UNC93B1 (3d mice) are highly susceptible to (*K*) *pneumoniae* infection. **(A)** Survival of WT, 3d, MyD88^-/-^, TLR4^-/-^ and TLR2^-/-^ mice after i.n. challenge with 1 x 10^7^ CFU of (*K*) *pneumoniae*. Shown are cumulative data from two experiments, each involving 8 animals per group. **, p<0.01; ***, p<0.001 versus WT mice, as determined by the log-rank test. **(B–E)** Log CFU numbers in the lungs or blood of WT, 3d, TLR4^-/-^, MyD88^-/-^ and TLR2^-/-^ mice at 24 and 48 h after i.n. infection. Horizontal red bars indicate mean values. The dashed lines indicate the limit of detection of the test. Each determination was conducted on a different animal in the course of two experiments, each involving 4 animals per group. ns, not significant, *, p<0.05; **, p<0.01; ***, p<0.001 versus WT mice as determined by the Mann-Whitney test.

To investigate the role of endosomal TLRs in immune sensing of *K. pneumoniae*, we used “triple-defective” (3d) mice carrying a missense mutation in UNC93B1, a chaperone protein responsible for trafficking and localization of endosomal TLRs. As a result, 3d mice are deficient in signaling through nucleic acid-sensing TLRs, such as TLR3, 7, 9, 11, and 13 (43). To assess the susceptibility of 3d mice to *K. pneumoniae*, we employed a pulmonary infection model in which bacteria were administered intranasally (i.n.) and replicated in the lungs. For comparison, we also included mice lacking MyD88, TLR4, or TLR2, as these molecules are known to contribute to anti-*K. pneumoniae* immune responses (27, 28, 44). Mice were challenged with a low, sublethal bacterial dose (as determined in preliminary experiments), and survival was monitored for up to 10 days. As expected, all wild-type (WT) mice survived without signs of disease. In contrast, MyD88-deficient mice displayed extreme susceptibility, with all succumbing within 3 days (**Figure 1A**). Although 3d mice survived longer than MyD88-deficient mice, they ultimately succumbed early during the infection, showing similar susceptibility to *K. pneumoniae* as TLR4-deficient mice. In comparison, TLR2-deficient mice exhibited greater resistance, with only 40% requiring humane euthanasia at late time points (**Figure 1A**). To determine whether increased lethality in immune-defective mice is due to a reduced ability to control bacterial replication, we assessed bacterial burden in the blood and lungs at 24 and 48 h post-challenge. At both time points, *K. pneumoniae* was detected in the lungs of all wild-type (WT) mice but only a few developed bacteremia (**Figures 1B–E**), indicating their ability to largely contain the infection within the lungs. In contrast, bacteria were detected in the blood of all 3d, MyD88-deficient, and TLR4-deficient mice, with significantly higher bacterial loads compared to WT animals (**Figures 1B–E**). These findings suggest that 3d mice are hypersusceptible to *K. pneumoniae* due to impaired control of bacterial replication in the lung and its spread to the bloodstream. Furthermore, the severity of the 3d phenotype is comparable to that of TLR4-deficient mice and more severe than that of TLR2-deficient mice.

### 3d mice are defective in proinflammatory responses to *K pneumoniae*

**Figure 2:**
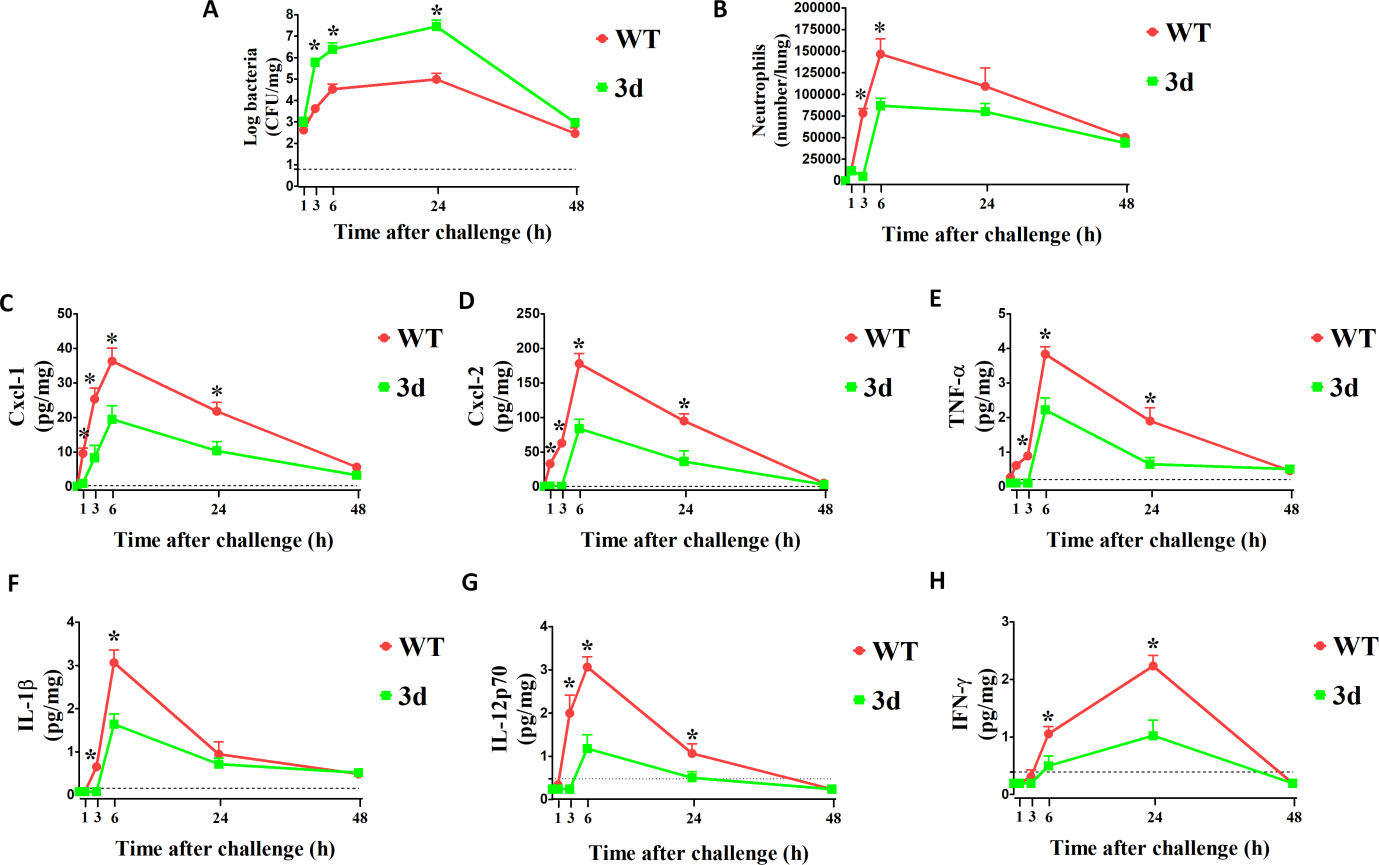
Mice with non-functional UNC93B1 (3d mice) are defective in neutrophil recruitment and cytokine responses during (*K*) *pneumoniae* pneumonitis. CFU numbers **(A)**, neutrophil counts **(B)** and cytokine concentrations **(C–H)** in the lungs at various times after i.n. challenge with 1 x 10^7^ CFU/mouse of (*K*) *pneumoniae*. Shown are results from one experiment involving 3 animals per time point. *, p<0.05 as determined by the Mann-Whitney test. Points and bars indicate means plus standard deviations. The dotted lines indicate the limits of detection of the tests.

Given their hypersusceptibility to *K. pneumoniae* infection, we next assessed the ability of 3d mice to mount defensive proinflammatory responses against the pathogen. Mice were challenged intranasally, as described previously, and cytokine concentrations, neutrophil infiltration, and bacterial burden were measured in lung homogenates. In wild-type (WT) mice, bacterial numbers steadily increased during the initial hours of infection, peaking at 24 hours and slowly declining thereafter (**Figure 2A**). In comparison, pathogen growth was faster in 3d mice, with bacterial numbers almost twice as high at 6 hours post-challenge. Neutrophil infiltration paralleled bacterial growth early during infection, peaking at 6 hours in both groups (**Figure 2B**). However, neutrophils influx was delayed and significantly reduced in 3d mice, despite higher bacterial loads. Neutrophils recruitment was associated with the production of several chemotactic and proinflammatory cytokines. As shown in **Figures 2C, D**, the neutrophil-attracting chemokines Cxcl1 and Cxcl2 were the first to increase above baseline in WT mice, being detectable as early as 1-hour post-challenge, whereas their production was delayed and diminished in 3d mice. Similarly, levels of TNF-α and IL-1β were lower in 3d mice early in infection (**Figures 2E, F**). Notably, both groups of mice exhibited a peak in IFN-γ at 24 hours, i.e. considerably later than other proinflammatory mediators (**Figures 2G, H**). Additionally, IL-12p70 and IFN-γ responses were more severely reduced in 3d mice compared to other cytokines. These findings suggest that endosomal receptors are critical for controlling *K. pneumoniae* infection in the lungs by promoting early chemotactic and proinflammatory cytokine production, as well as neutrophil recruitment. Furthermore, these receptors play a particularly important role in mediating IL-12p70 and IFN-γ responses to *K. pneumoniae*.

### Endosomal TLRs promote macrophage responses to *K pneumoniae*

**Figure 3:**
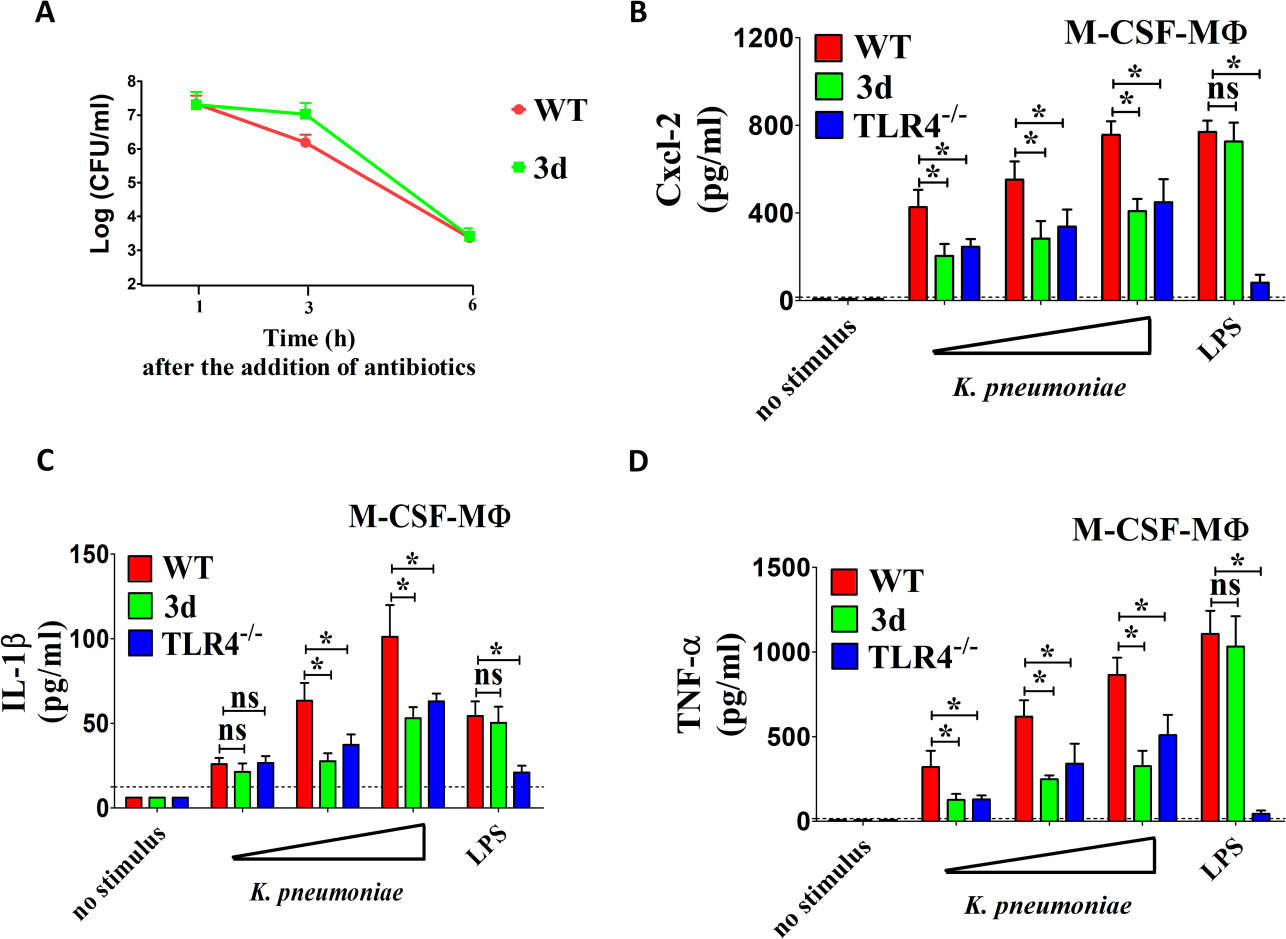
Endosomal TLRs participate in chemokine and cytokine responses to *K. pneumoniae* by M-CSF polarized macrophages. **(A)** Bacterial killing assay in bone marrow macrophages cultured in the presence of M-CSF obtained from wild type (WT) and 3d mice. Cells (5x10^5^) were exposed to *K. pneumoniae* (1x10^7^ CFU) for 1 h at 37°C in 5% CO_2_. Extracellular bacteria were then killed through the addition of gentamicin (100 µg/ml), and cultures were further incubated for 1, 3, and 6 h before determination of CFU numbers in cell lysates. Shown are means plus standard deviations from three experiments conducted in triplicate. Statistical analysis was conducted by the Mann-Whitney test. **(B–D)** Cxcl2, IL-1β and TNF-α concentrations in 24-h culture supernatants of bone marrow macrophages cultured in the presence of M-CSF (M-CSF-MΦ) isolated from WT, TLR4^-/-^ or 3d mice and stimulated with increasing amounts (MOIs of 5, 10, and 20) of bacteria or with LPS (0.1 µg/ml). ns, not significant; *, p<0.05 as determined by the Mann-Whitney test. The dotted lines indicate the limits of detection of the tests.

Given the significant reduction in neutrophil infiltration and cytokine production observed during *in vivo* infection, we sought to investigate *in vitro* cytokine responses to *K. pneumoniae* in macrophages, as these cells are key producers of inflammatory mediators. To this end, we cultured WT and 3d bone marrow-derived macrophages in the presence of macrophage colony-stimulating factor (M-CSF-MΦ) and assessed their bactericidal activity and inflammatory mediator production. 3d M-CSF-MΦ displayed no significant defects in bacterial internalization or intracellular killing compared to WT M-CSF-MΦ (**Figure 3A**). However, 3d cells were significantly, albeit partially, impaired in their ability to release Cxcl2, IL-1β and TNF-α in response to *K. pneumoniae* compared to WT cells (**Figures 3B–D**). These defects were specific for stimulation with whole bacteria, since 3d macrophages exhibited normal responses to LPS, a well-known TLR4 agonist. Moreover, the extent of the reduction in pro-inflammatory cytokine production in 3d macrophages was similar to that observed in cells deficient in TLR4, which is known to play a crucial role in immune sensing of Gram-negative bacteria, including *K. pneumoniae*.

**Figure 4:**
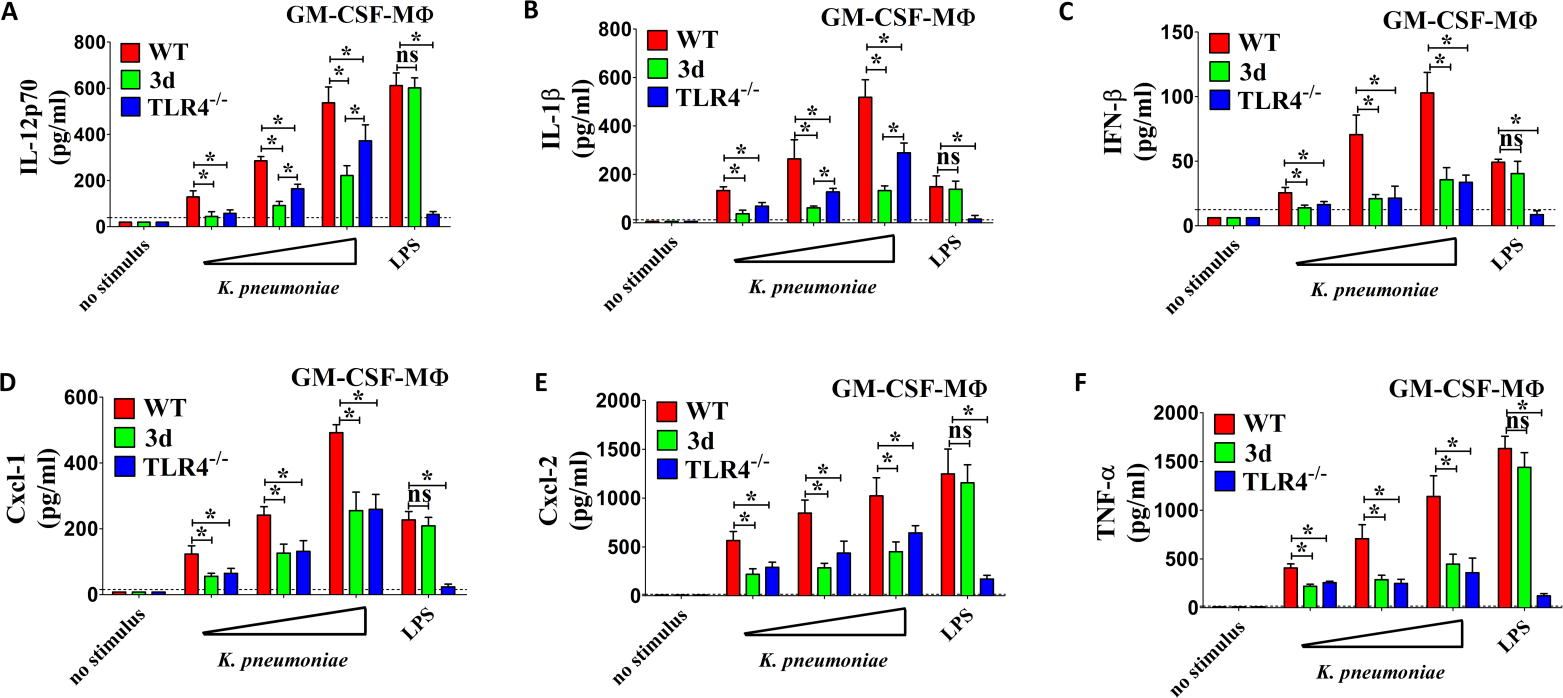
Cytokine responses to *K. pneumoniae* in GM-CSF polarized macrophages. **(A–F)** IL-12p70, IL-1β, IFN-β, Cxcl1, Cxcl2 and TNF-α protein levels in 24-h culture supernatants of macrophages (GM-CSF-MΦ) obtained from the bone marrows of WT, TLR4^-/-^ or 3d mice, cultured in the presence of GM-CSF and stimulated with increasing amounts (MOI of 5, 10, and 20) of bacteria or with LPS (0.1 µg/ml). Shown are means plus standard deviations from three experiments conducted in triplicate. *, p<0.05 as determined by the Mann-Whitney test. The dotted lines indicate the limits of detection of the tests.

In view of the important role of GM-CSF in macrophage differentiation, we also used bone marrow-derived MΦ cultured in the presence of this growth factor. Notably, while WT GM-CSF-MΦ produced high levels of IL-12p70, IL-1β and IFN-β in response to *K. pneumoniae*, the production of IL-12p70 and IL-1β was markedly impaired in 3d GM-CSF-MΦ, with the extent of reduction surpassing that observed in TLR4-deficient macrophages. (**Figures 4A. B**). However, the levels of IFN-β, Cxcl1, Cxcl2 and TNF-α were decreased to a similar degree in 3d and TLR4-KO MΦ (**Figures 4C–F**). All together, these data revealed that endosomal TLRs are crucial for the production of pro-inflammatory cytokines, particularly IL-12p70 and IL-1β, by bone marrow derived macrophages stimulated with *K. pneumoniae*. Furthermore, the presence of TLR4 cannot compensate for this defect.

**Figure 5:**
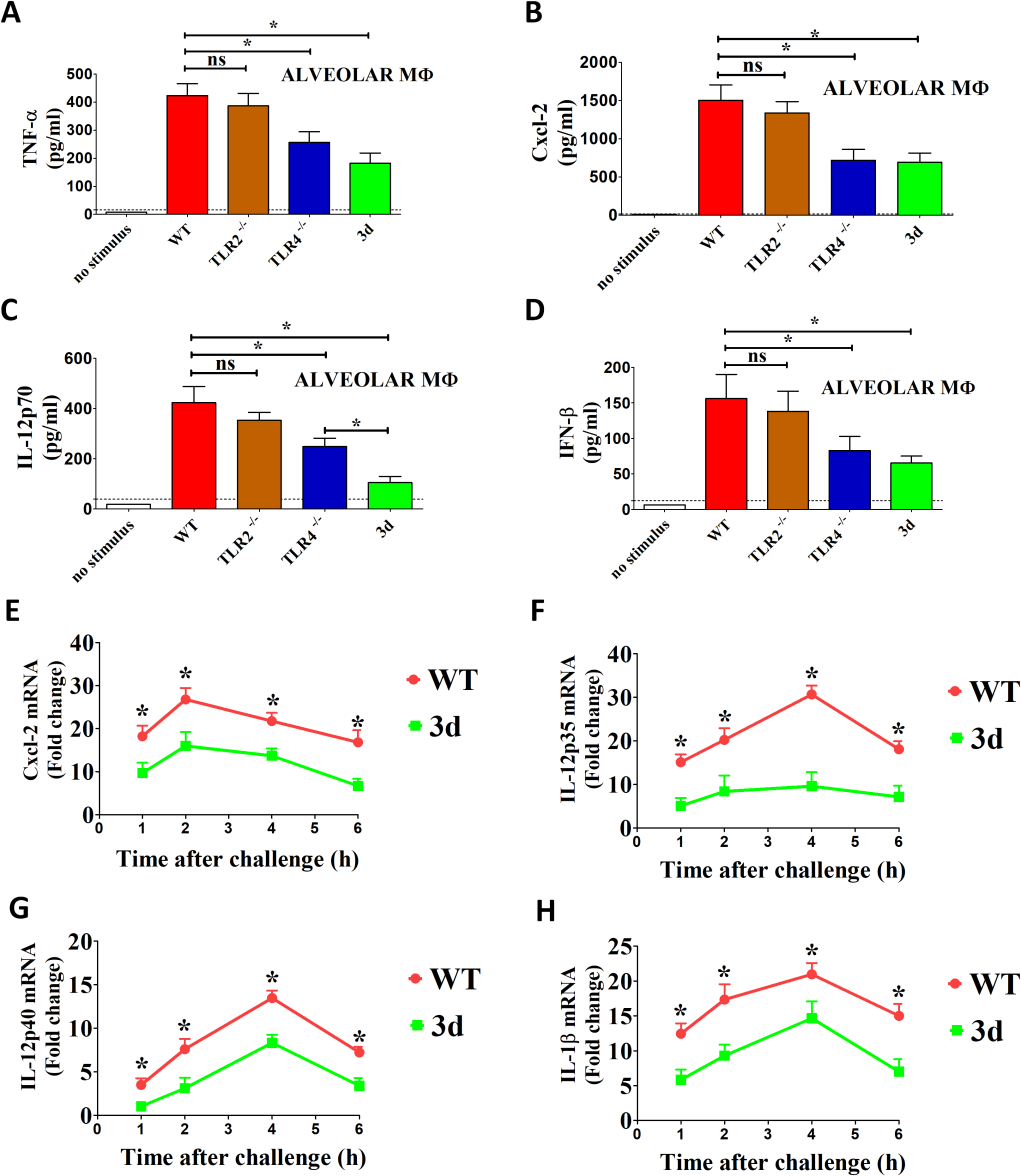
Role of endosomal TLRs in cytokine response to *K. pneumoniae* by alveolar macrophages. **(A–D)** Alveolar macrophages (MΦ) from WT, TLR4^-/-^ or 3d mice were stimulated with *K. pneumoniae* (MOI of 10), and cytokine concentrations were measured in 24-h culture supernatants. The dotted lines indicate the limits of detection of the tests. Means ± SD from three independent experiments conducted in triplicate. ns, not significant, *, p<0.05 as determined by the Mann-Whitney test. The dotted lines indicate the limits of detection of the tests. **(E–H)** RT-qPCR assessment of cytokine mRNA levels in GM-CSF-polarized bone marrow macrophages at different times after stimulation with *K. pneumoniae* (MOI of 10). *, p<0.05 as determined by the Mann-Whitney test.

In subsequent experiments, we investigated the role of endosomal TLRs in the recognition of *K. pneumoniae* by alveolar macrophages (AM), given the critical role that these cells play in immune defenses against pulmonary infections. Consistent with our previous findings in bone marrow-derived macrophages, the levels of TNF-α, Cxcl2, IL-12p70, and IFN-β were significantly reduced in 3d AM following stimulation with *K. pneumoniae*. These results suggest that nucleic acid-sensing TLRs play a key role in the response of alveolar macrophages to *K. pneumoniae* infection (**Figures 5A–D**). To explore whether these effects are linked to transcriptional regulation, we assessed mRNA levels for various cytokines in AM stimulated with *K. pneumoniae* (**Figures 5E–H**). Cytokine mRNA levels increased significantly within the first few hours after bacterial stimulation, peaking at 2-4h post-infection. While IL-12p40, IL-1β, and Cxcl2 mRNA levels were only moderately impaired in 3d AM, IL-12p35 mRNA expression was markedly reduced, suggesting that the pronounced decrease in IL-12p70 levels observed in AM supernatants is secondary to a selective reduction in IL-12p35 mRNA transcription (**Figure 5F**).

**Figure 6:**
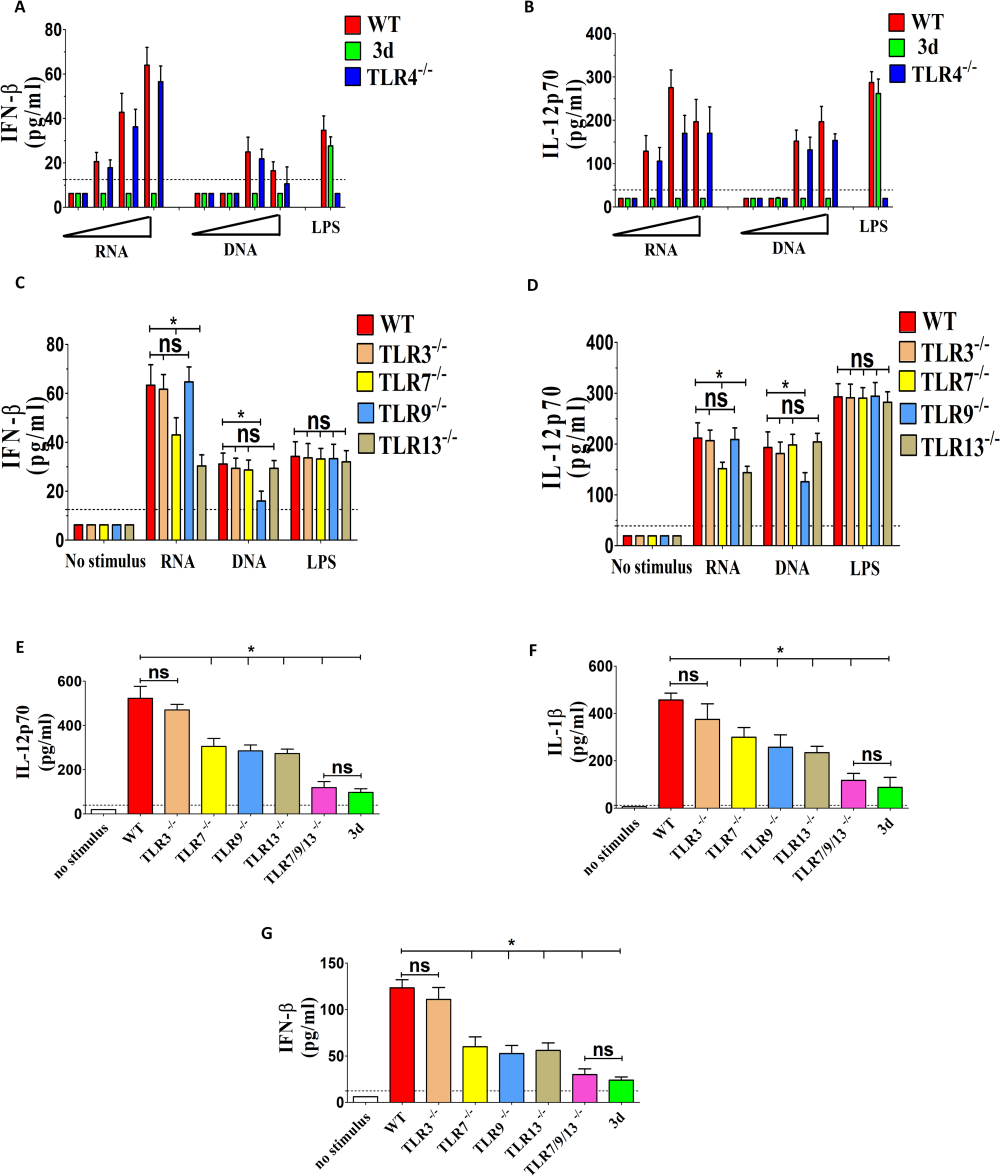
RNA from *K. pneumoniae* induces cytokine production by activating endosomal TLRs in GM-CSF-polarized bone marrow macrophages. **(A–D)** Production of IFN-β and IL-12p70 in GM-CSF-polarized bone marrow macrophages obtained from the indicated mouse strains and stimulated with graded doses (0.01, 0.1, 1 and 10 μg/ml; **A**, **B**), or with 10 μg/ml **(C, D)** of purified DNA or RNA from *K. pneumoniae*. DNA and RNA were complexed with N-[1-(2,3-dioleoyloxy) propyl]-N, N, N-trimethylammonium methyl-sulfate (DOTAP) before cell stimulation. **(E–G)** Production of IL-12p70, IL-1β and IFN-β in GM-CSF-polarized bone marrow macrophages obtained from the indicated mouse strains and stimulated with *K. pneumoniae* (MOI 10). In all experiments, cytokine concentrations were determined in culture supernatants obtained at 24 h after stimulation, using *E. coli* LPS (0.1 μg/ml) as a positive control. The dotted lines indicate the limits of detection of the tests. Data are expressed as means ± SD of three independent experiments conducted in duplicate. *, p<0.05; as determined by the Mann-Whitney test. ns, not significant.

Overall, impaired cytokine gene transcription and increased susceptibility to infection in the absence of nucleic acid-sensing endosomal receptors suggest a role for *K. pneumoniae* DNA and/or RNA in stimulating innate immune responses. To gain further insights, we purified *K. pneumoniae* DNA and RNA and used them to stimulate GM-CSF-polarized macrophages. Both nucleic acid types could induce the release of IFN-β and IL-12p70 from these cells in a dose-dependent manner, with RNA showing significantly higher potency (**Figures 6A, B**). Responses to nucleic acids, but not to LPS, were completely abrogated in 3d macrophages. Moreover, GM-CSF-polarized macrophages lacking TLR7 or TLR13 were partially unable to respond to RNA, while macrophages lacking TLR9 were defective in their ability respond DNA (**Figures 6C, D**).

To assess the contribution of bacterial RNA to the overall ability of whole *K. pneumoniae* to induce cytokine production, we tested macrophages from mice lacking one or more RNA-sensing TLRs (TLR3, 7 and 13) in comparison to the DNA-sensing receptor TLR9. Moderate, though significant, reductions in IL-12p70, IL-1β and IFN-β responses to *K. pneumoniae* were observed in GM-CSF-MΦ from mice lacking TLR7, 9 or 13, but not in cells lacking TLR3 (**Figures 6E–G**). However, such responses were almost completely abrogated in triple-KO cells simultaneously lacking TLR7, 9 and 13, thereby phenocopying 3d cells (**Figures 6E–G**). Collectively, these data indicate that *K. pneumoniae* nucleic acids are potent inducers of proinflammatory cytokines, particularly IL-12p70, in macrophages, and that recognition of bacterial RNA through TLR7 or TLR13 contributes to these responses, similar to TLR9-mediated DNA recognition.

### Endosomal TLRs in neutrophils promote ROS release in response to *K pneumoniae*

**Figure 7:**
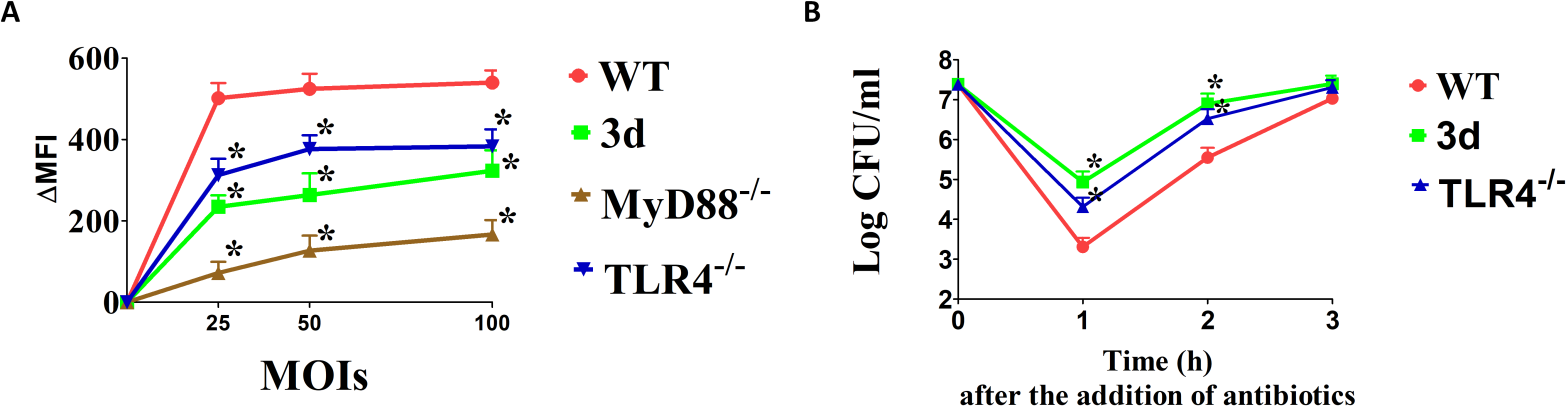
Endosomal TLRs are required for optimal (*K*) *pneumoniae* killing. **(A)** Fluorescence intensities (ΔMFI) of bone marrow derived neutrophils obtained from WT, TLR4^-/-^ or 3d mice stimulated with the indicated MOIs of (*K*) *pneumoniae* and examined by fluorescence flow cytometry using the CellROX fluorescent reagent. Data are expressed as means ± SD of three independent experiments conducted in duplicate. *, p<0.05 as determined by Mann-Whitney test. **(B)** Killing of (*K*) *pneumoniae* by bone marrow-derived neutrophils from WT, TLR4^-/-^ or 3d mice. Cells were exposed to (*K*) *pneumoniae* (1x10^5^ CFU/well) and bacterial viability was determined at the indicated time points. Data are expressed as means ± standard deviations of three independent experiments conducted in duplicate. *, p<0.05 as determined by the Mann-Whitney test.

Defective reactive oxygen species (ROS) production has been linked to hypersusceptibility to *K. pneumoniae* (45). Given the crucial role of neutrophils in bacterial killing, we investigated whether neutrophils lacking endosomal TLRs are impaired in their ability to produce ROS in response to *K. pneumoniae*. Bone marrow-derived neutrophils were exposed to increasing concentrations of *K. pneumoniae*, and ROS production was assessed using a fluorescence flow cytometry assay. Under these conditions, *K. pneumoniae* induced ROS production, which was primarily dependent on TLR signaling, as evidenced by an over 80% reduction in ROS levels in neutrophils lacking the TLR adaptor MyD88. Notably, neutrophils from 3d or TLR4-deficient mice were also significantly impaired in their ROS production (**Figure 7A**). Additionally, bactericidal activity was significantly, though moderately, reduced in 3d and TLR4-deficient neutrophils (**Figure 7B**). Collectively, these data indicate that TLRs, and in particular endosomal TLRs and TLR4, play an important role in neutrophil ROS production and in the ability of these cells to kill *K. pneumoniae*.

## Discussion

Considerable efforts have been made in recent years to understand the mechanisms underlying innate immune responses to *K. pneumoniae* infections, as this knowledge is crucial for elucidating the pathogenesis of the disease and developing alternative therapeutic strategies (46). Previous studies have highlighted the central role of TLRs in *K. pneumoniae* recognition with marked susceptibility to infection observed in the absence of TLR adaptors such as MyD88, TRIF, and TIRAP (47, 48). While the roles of a few isolated receptors (including TLR2, TLR4, and TLR9) have been explored, the relative contributions of these and other TLRs to the host’s overall ability to control infection, as well as the potential interactions between these receptors, remain poorly understood. In particular, the role of nucleic acid-sensing TLRs in defense against *K. pneumoniae* have not been fully examined, with only a few studies addressing this aspect (20, 22, 49). Curiously, while the absence of TLR9 impairs host defenses to a lesser extent than the absence of TLR4 (20), the lack of TLR3 paradoxically enhances bacterial clearance via a macrophage-dependent mechanism (46).

Here we show that when the function of multiple nucleic acids sensing TLRs is compromised —such as in 3d mice with a loss-of-function mutation in the UNC93B1 chaperone— anti-*K. pneumoniae* host defenses are markedly impaired. This phenotype is as severe as that observed in TLR4-KO mice and nearly as severe as in MyD88-deficient mice. Notably, the increased lethality observed in all these mouse strains is linked to an impaired ability to produce optimal levels of chemotactic and pro-inflammatory cytokines *in vivo*. This deficiency leads to defective neutrophil recruitment to the lungs, uncontrolled pathogen growth, and systemic bacterial dissemination. The reduced cytokine levels observed *in vivo* were mirrored by reduced cytokine production by both bone marrow derived and alveolar macrophages infected *in vitro*. However, the contribution of other cell types (such as epithelial cells) cannot be ruled out and this requires further investigation.

Interestingly, we found that 3d macrophages are more severely impaired than TLR4 KO macrophages in their ability to produce IL-12p70 in response to *K. pneumoniae*, although both cell types show similar deficits in producing other cytokines. The defective IL-12p70 release by 3d macrophages was linked to impaired activation of the gene encoding the IL-12p35 subunit, which may be associated with selective targeting of this gene by nucleic acid-sensing TLRs through the activation of IFN regulatory transcription factors 1 and 5 (30, 50, 51). IL-12p70 is primarily produced by mononuclear phagocytes and plays a key role in inducing IFN-γ, a critical antibacterial factor, in group 1 innate lymphoid cells and T lymphocytes (49). In this study, IFN-γ peaked significantly later than IL-12p70 in the lungs of infected animals, suggesting that the reduced IFN-γ levels observed in 3d mice may, at least in part, be due to defective IL-12p70 production by mononuclear phagocytes early during infection.

Notably, macrophages lacking isolated endosomal receptors such as TLR3, 7, 9 and 13 exhibited no or only slight defects in cytokine production, suggesting that these receptors can compensate for each other’s absence. This redundancy may have evolved to prevent pathogens from evading immune detection. However, the combined loss of TLR7, 9 and 13 resulted in severely impaired cytokine production, indicating that the presence of cell surface receptors (e.g. TLR4 and TLR2) and cytosolic innate immune sensors cannot compensate for lack of endosomal TLR function. Indeed, the absence of TLR2 had no effect on cytokine responses to *K. pneumoniae* and was associated with only a slight increase in lethality late during infection, consistent with previous studies (26). Thus, while confirming the fundamental importance of LPS recognition by TLR4 in *K. pneumoniae* sensing, we demonstrate here that an equally essential role is played by a complementary endosomal receptor system by which TLR7, 9 and 13 cooperate in detecting the presence of different forms of *K. pneumoniae* nucleic acids, likely released by bacterial digestion in phagolysosomes (30).

As mentioned above, triple KO cells simultaneously lacking TLR7, 9 and 13 produced much lower cytokine levels than those lacking only TLR9. This suggests that, in addition to the DNA-sensing TLR9, RNA-sensing TLR7 and 13 play significant roles in cytokine responses against *K. pneumoniae*, as supported by the potent immune-stimulating effects of bacterial RNA compared to DNA. Collectively, our data highlight the significant role of prokaryotic RNA in *K. pneumoniae* immune sensing, extending to Gram-negative bacteria similar findings previously observed with pyogenic Gram-positive cocci (30, 32, 33, 52–54). These results lend support to the emerging view that RNA is an important target of bacterial recognition even in pathogens where LPS has traditionally been considered the dominant immune-activating signal. For example, blockade of the RNA sensing TLR8 receptor significantly impaired the production of IL-12p70 and IL-1β in human monocytes infected with *P. aeruginosa* (51). It should be noted, in this respect, that human TLR8 plays an important role in recognizing pathogens that are sensed predominantly by TLR13 in murine systems, as TLR13 is absent in humans, while TLR8 is almost completely nonfunctional in mice (31–33, 55, 56). Finally, we reveal a novel function of TLRs in anti-*K. pneumoniae* defenses, linked to the ability of TLR4 and endosomal TLRs to enhance ROS production and bactericidal activity in neutrophils. These findings align with the emerging view that TLRs can promote ROS responses to bacteria (13, 14).

In conclusion, our data demonstrate that defective endosomal receptor function leads to hypersusceptibility to *K. pneumoniae* infection, primarily due to impaired cytokine production by macrophages, reduced neutrophil recruitment and defective bacterial killing by neutrophils. Future studies will investigate the contributions of other cell types, such as epithelial cells and innate lymphoid cells, to TLR-dependent anti-*K. pneumoniae* responses. Of note, our findings also highlight the significant role of RNA as a target for innate immune recognition of *K. pneumoniae*. These insights may prove valuable in developing strategies to stimulate these receptors, offering potential therapeutic avenues for treating infections caused by antibiotic-resistant strains.

## Data Availability

The authors acknowledge that the data presented in this study have been deposited and are publicly available at the following links https://figshare.com/s/cb9e1c04eb7d44b7d50a and doi: 10.6084/m9.figshare.28677941.
